# Engagement With mHealth COVID-19 Digital Biomarker Measurements in a Longitudinal Cohort Study: Mixed Methods Evaluation

**DOI:** 10.2196/40602

**Published:** 2023-01-13

**Authors:** Kirsten L Rennie, Emma R Lawlor, Arrash Yassaee, Adam Booth, Kate Westgate, Stephen J Sharp, Carina S B Tyrrell, Mert Aral, Nicholas J Wareham

**Affiliations:** 1 Medical Research Council Epidemiology Unit University of Cambridge Cambridge United Kingdom; 2 Huma Therapeutics Limited London United Kingdom

**Keywords:** smartphone, apps, engagement, COVID-19, pandemic, cohort studies, epidemiology, mobile health, digital health, biomarker, mobile phone

## Abstract

**Background:**

The COVID-19 pandemic accelerated the interest in implementing mobile health (mHealth) in population-based health studies, but evidence is lacking on engagement and adherence in studies. We conducted a fully remote study for ≥6 months tracking COVID-19 digital biomarkers and symptoms using a smartphone app nested within an existing cohort of adults.

**Objective:**

We aimed to investigate participant characteristics associated with initial and sustained engagement in digital biomarker collection from a bespoke smartphone app and if engagement changed over time or because of COVID-19 factors and explore participants’ reasons for consenting to the smartphone substudy and experiences related to initial and continued engagement.

**Methods:**

Participants in the Fenland COVID-19 study were invited to the app substudy from August 2020 to October 2020 until study closure (April 30, 2021). Participants were asked to complete digital biomarker modules (oxygen saturation, body temperature, and resting heart rate [RHR]) and possible COVID-19 symptoms in the app 3 times per week. Participants manually entered the measurements, except RHR that was measured using the smartphone camera. Engagement was categorized by median weekly frequency of completing the 3 digital biomarker modules (categories: 0, 1-2, and ≥3 times per week). Sociodemographic and health characteristics of those who did or did not consent to the substudy and by engagement category were explored. Semistructured interviews were conducted with 35 participants who were purposively sampled by sex, age, educational attainment, and engagement category, and data were analyzed thematically; 63% (22/35) of the participants consented to the app substudy, and 37% (13/35) of the participants did not consent.

**Results:**

A total of 62.61% (2524/4031) of Fenland COVID-19 study participants consented to the app substudy. Of those, 90.21% (2277/2524) completed the app onboarding process. Median time in the app substudy was 34.5 weeks (IQR 34-37) with no change in engagement from 0 to 3 months or 3 to 6 months. Completion rates (≥1 per week) across the study between digital biomarkers were similar (RHR: 56,517/77,664, 72.77%; temperature: 56,742/77,664, 73.06%; oxygen saturation: 57,088/77,664, 73.51%). Older age groups and lower managerial and intermediate occupations were associated with higher engagement, whereas working, being a current smoker, being overweight or obese, and high perceived stress were associated with lower engagement. Continued engagement was facilitated through routine and personal motivation, and poor engagement was caused by user error and app or equipment malfunctions preventing data input. From these results, we developed key recommendations to improve engagement in population-based mHealth studies.

**Conclusions:**

This mixed methods study demonstrated both high initial and sustained engagement in a large mHealth COVID-19 study over a ≥6-month period. Being nested in a known cohort study enabled the identification of participant characteristics and factors associated with engagement to inform future applications in population-based health research.

## Introduction

### Background

Mobile health (mHealth) apps are increasingly being used in health research with the rapid uptake of smartphones and availability of digital tools enabling measurement of vital signs and biomarkers outside a clinical or research facility. The COVID-19 pandemic has accelerated the interest in developing and implementing mHealth in both health care and health research [1-3]. These systems can be used for passive data collection, where continuous or episodic data are automatically uploaded from integrated devices and wearables, or active data collection, where participants need to interact with the system to either complete short questionnaires or log measurements from additional devices [4].

The use of mHealth systems in patient studies is well documented, supporting patient monitoring in chronic conditions, acute COVID-19 [5], and the conduct of clinical trials [6]. In contrast, there are limited reports of where these systems have been used to support population-based research outside specific patient groups. Readily accessible mHealth platforms that facilitate easy user-led data entry could enable cost-effective longitudinal research to be conducted on a much larger scale with more frequent data collection than currently used in population-based studies. If studies can be conducted entirely remotely with no need for in-person recruitment or visits to standard research clinic settings, this could potentially increase the size and reach of population-based research, making participation more accessible to some groups that could have been difficult to recruit previously for in-person research visits.

A recent systematic review assessing engagement with mHealth technology reported that studies to date have been relatively short and with small sample sizes, with a lack of use and acceptability evidence both from quantitative and qualitative data [7]. Specifically, there is a lack of evidence on engagement and adherence in nonpatient groups, which are not driven to engage with mHealth systems because of a medical need, particularly evaluating barriers and enablers that can inform future health equity considerations in research.

Epidemiological studies that have used mHealth systems in nonpatient populations have often been limited by the length of the study (≤3 months), relatively small sample sizes, and the requirement of an in-person component to enroll and set up the app, and have often only included participants with iOS devices [8,9]. There is a need to understand how participants engage with mHealth platforms at the outset—the initial engagement with the system—and a longer sustained engagement and whether there may be important selection bias beyond that observed in traditional epidemiological studies.

### Objectives

We conducted a fully remote population-based study using a smartphone app nested within an existing, established longitudinal cohort, the Fenland study in healthy middle-aged and older adults. The objective of the app substudy was to understand the natural history of COVID-19 from the presymptomatic stage to the symptomatic stage by tracking digital biomarkers and a symptom log using active data collection 3 times per week.

The objectives of this engagement study were to (1) investigate participant characteristics associated with initial engagement in the app substudy and compare with those who did not consent to the substudy, (2) assess participant characteristics associated with engagement with the app measurements, (3) assess if participant continued engagement declined or changed over time and if engagement was associated with factors such as national COVID-19 social restriction levels or experiencing symptoms of potential COVID-19, and (4) explore participants’ reasons for consenting (or not) to the app substudy and experiences related to initial and continued app engagement.

## Methods

### Participant Recruitment: The Fenland Cohort

Participants were recruited from an existing cohort, the Fenland study. The cohort study was established in 2005, recruiting participants from primary care registers across Cambridgeshire who were born between 1950 and 1975 [10]. Phase 2 clinical visits started in 2014 and were halted at the start of the COVID-19 pandemic in the United Kingdom in March 2020. Further information on the Fenland cohort and exclusion criteria is provided in Multimedia Appendix 1 [11-16].

### Participant Recruitment: Fenland COVID-19 Study

**Figure 1:**
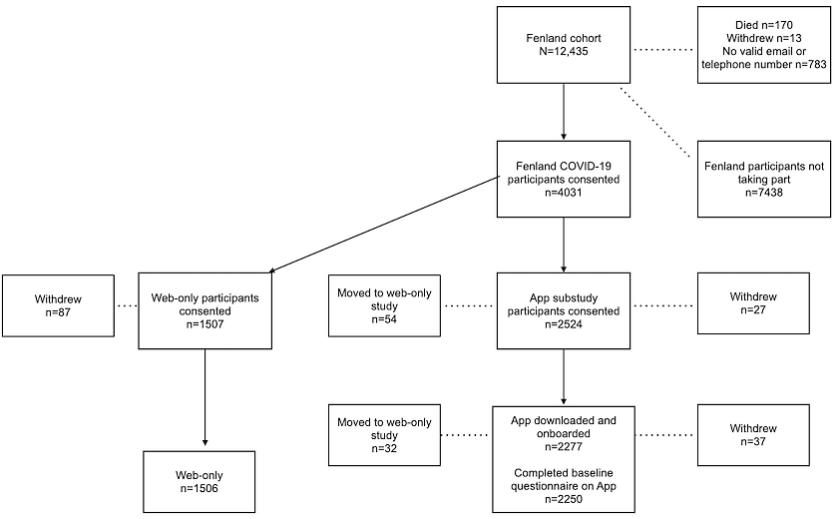
Fenland COVID-19 study flow diagram.

The main aim of the COVID-19 study was to determine the prevalence of previous infection with COVID-19 in this known population-based study using 3-monthly blood sample measures of SARS-CoV-2 immunoglobulin G (IgG) antibodies [11]. The study was designed as an observational cohort with data collection for 6 months with the option of extending for a further 6 months depending on the COVID-19 pandemic. Participants were informed that the study would be for a minimum of 6 months and would be extended if it were deemed to be informative for public health. Fenland cohort study participants who had not died or withdrawn and had a valid telephone number or email address (n=11,469) were invited via email, SMS text message, or telephone call to take part in the Fenland COVID-19 study from July 2020 onward (Figure 1). All participants who took part in the main Fenland cohort study were eligible to take part in this study, with no exclusion criteria applied. Participants were provided with information on the study and completed web-based consent remotely [17].

### Participant Recruitment: Fenland-Huma App Substudy

After consenting into the Fenland COVID-19 study, participants were sent further information on the app substudy, which was designed in collaboration with Huma, a digital health company specializing in remote patient monitoring [18]. The study objective was to understand the natural history of COVID-19 by tracking digital biomarkers, symptoms, and other self-reported health information within the Huma app together with the COVID-19 antibody blood results.

To improve the external validity of the findings for future remote population research, the app substudy was conducted using an entirely remote setup with no option of in-person configuration or onboarding. Aside from activity data from smartphone-based step count, all measurements included in this engagement study required patients to manually input their results into the app.

Eligible participants required a smartphone with either software version iOS 13.0 or above or Android 6.0 or above. If participants agreed to take part in the substudy, they were sent a link to a second web-based consent form. Participants were posted a digital pulse oximeter (ChoiceMMed MD300C29) and a sublingual thermometer (Genial Digital Thermometer T12L) with instructions for use. Participants were emailed a link to download the app from the Google Play Store or Apple App Store together with a unique participant identifier and password to securely log into the Fenland COVID-19 Huma app and directed to complete a baseline questionnaire. Further information on how to download and start using the app (downloading the app, app consent, and registering) was provided on the study website and via a helpline (email and telephone) if required. Technical support was given throughout the study via in-app support or via email. The app included links to further information on the study website and the helpline.

Participants consented into the study from July 5, 2020, onward, and those in the app substudy were able to download and onboard to the app from August 6, 2020, onward. Owing to the timing of the UK rollout of SARS-CoV-2 vaccines in this age group, it was decided by the research project team to end the data collection period once all participants had reached the 6-month blood sample collection time point to measure SARS-CoV-2 IgG antibodies. The study finished on April 30, 2021, and the app closed to further measurement entry.

### App Modules

**Figure 2:**
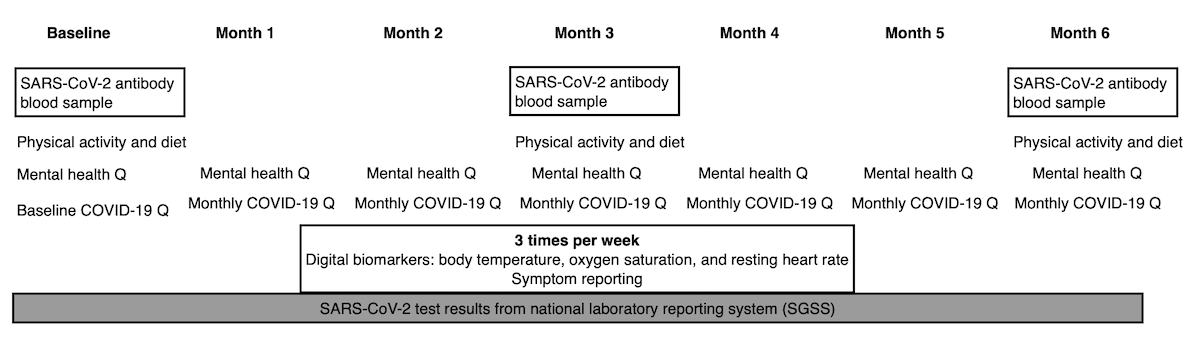
Fenland COVID-19 web and app study measurements. Q: questionnaire; SGSS: Second Generation Surveillance System.

**Figure 3:**
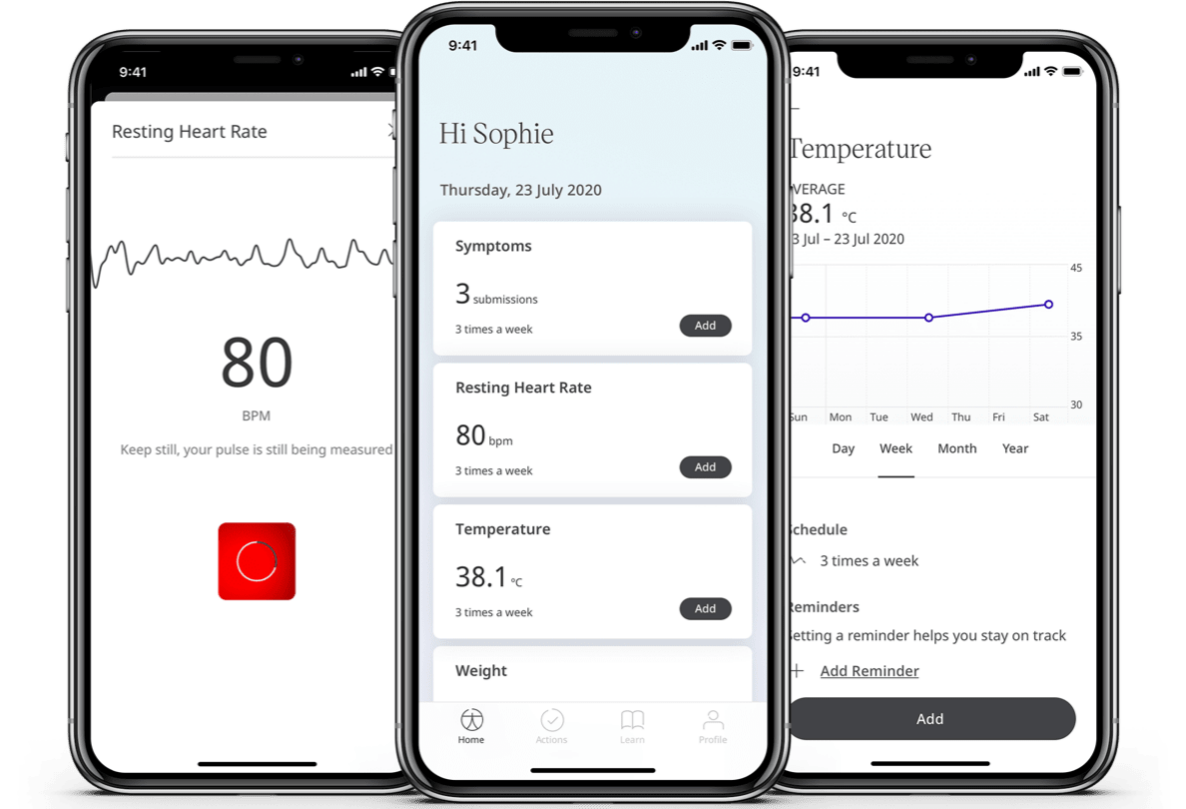
Screenshots of the Fenland COVID-19 study app interface with resting heart rate measurement, home screen, and temperature measurement.

The Huma app was configured for tailored data entry modules that required measurements to be entered at differing frequencies (Figures 2 and 3). The frequency of the measurements was determined in relation to the current evidence on the etiology of SARS-CoV-2 and the time frame of the validated measure and in consultation with the Fenland Participant and Public Involvement (PPI) panel to minimize participant burden.

Participants received automated prompts on their phones to remind them to complete the monthly questionnaires. For the digital biomarker and symptom modules, participants were able to set their own time reminders and were provided with instructions on how to do this.

The app content was tested with a panel group within the Medical Research Council Epidemiology Unit to ensure that the questions, instructions, and accompanying “Learn” sections were clear and unambiguous before the app content was finalized and launched.

### COVID-19 Digital Biomarker and Symptom Modules

Participants were asked to complete 4 measurement modules 3 times per week: oxygen saturation, body temperature, resting heart rate (RHR), and symptom recording. Engagement in completing these 4 modules during the study period was used as the main outcome for this study. For reasons of practicality and to control for diurnal variation, participants were asked to take all measurements first thing in the morning after awaking.

Participants used the digital thermometer and pulse oximeter provided to measure their body temperature and oxygen saturation, respectively, and were asked to manually enter the results into the app. Participants were asked to measure their RHR using photoplethysmography within the app by placing their finger over the camera on their smartphone. The measurement took approximately 60 seconds to provide a measure of RHR [19]. They were also asked to record whether they were experiencing any symptoms from a list provided or select the option “no symptoms.” They were also asked to record whether they were experiencing any symptoms from a list provided or select the option “no symptoms.” Participants could select as many symptoms as were applicable to them by clicking on the symptom. The option to add other symptoms not in the list was given. The list of symptoms was updated regularly during the study as further symptoms were reported by a substantial proportion of participants or as new public health information on symptoms emerged. For this analysis, symptoms for each entry were categorized as yes or no. It was estimated that it would take approximately 6 minutes to complete these 4 modules per day. Further instructions on how to take these measurements in written or video form were provided on the study website, and links were given in the “Learn” section on the app.

### Sociodemographic Characteristics

The participants’ current postcode at the start of the Fenland COVID-19 study was used to derive the English indices of multiple deprivation 2019 and the 2011 rural-urban classification [20,21].

Educational attainment (degree level), ethnicity, and occupation were self-reported during phase 1 of the Fenland study. Occupation was categorized into 3 occupation groups [22]. Group 1 was routine manual and service, semiroutine, and technical; group 2 was middle or junior managers, clerical, and intermediate; and group 3 was traditional professional, modern professional, and senior managers [23].

A baseline questionnaire was completed within the app by those in the app substudy. Regarding those who did not take part in the app study, the same baseline questionnaire was completed on a web-based form. Participants reported their living situation: living alone, with family, or with a partner or friends or living with a carer or being a live-in carer. Owing to very low numbers in the latter 2 categories, these were collapsed into an “other” category.

### Self-reported Health Characteristics

Participants also completed questions on smoking habits, self-reported health quality, existing health conditions, physical function, and body weight in the baseline questionnaire.

Physical activity energy expenditure over the previous 4 weeks was determined using the electronic web-based version of the validated Recent Physical Activity Questionnaire [24]. Working status category was derived from the Recent Physical Activity Questionnaire from those who reported that they were working. The baseline questionnaire–derived physical activity energy expenditure was used in this study.

### Mental Health

Measures of depression, anxiety, and perceived stress were made throughout the study period on the bespoke app and completed by participants in the app substudy only. In this study, we used the baseline measurements. Depression was measured using the Patient Health Questionnaire-8 [14] to define current depression [14,15]. Anxiety was measured using the Generalized Anxiety Disorder questionnaire [13]. Perceived stress was measured using the Perceived Stress Scale [16].

### Antibody Status to SARS-CoV-2

Dried blood spot samples collected remotely by participants were analyzed for SARS-CoV-2 IgG antibodies [11]. Results were classified into 2 categories: positive or negative (negative or borderline results). Baseline IgG antibody results were used to ascertain whether exposure to SARS-CoV-2 influenced initial engagement level. Results from all antibody tests during the study were also combined to determine if a positive antibody test was associated with continued engagement. Participants were categorized as positive if any result was positive at any of the 3 time points (0, 3, and 6 months) or as negative if the result was not positive at any of the 3 time points.

### COVID-19 Infection Test Results

SARS-CoV-2 testing data from the Second Generation Surveillance System, the national reporting system across England, were obtained for all Fenland COVID-19 study participants during the study period. These contained all routine laboratory tests for SARS-CoV-2 infections from hospitals (patients and National Health Service key workers) and community testing in the general population before and during the study period. In this analysis, we used the first confirmed positive polymerase chain reaction test result to classify if participants had a COVID-19 infection either before or during the study using the date of the polymerase chain reaction test.

### Engagement Outcomes

The study period for each participant was calculated from the date the baseline questionnaire was completed until one of the following: (1) the end of the study period (April 30, 2021), (2) the date the participant contacted the study team to withdraw from the study, or (3) the date the participant contacted the study team to withdraw from the app substudy. The number of complete study weeks was calculated for each participant. For the COVID-19 digital biomarker and symptom modules, the frequency of module entries for each study week was calculated. As participants had been asked to complete these modules 3 times per week, 3 engagement categories were generated for each study week: 0 (no entries), 1 to 2 times per week, and ≥3 times per week.

### National COVID-19 Social Restriction Levels

Restriction severity was categorized as (1) minimal restrictions (schools open but social gathering restrictions still in place; June 1, 2020-November 4, 2020), (2) moderate restrictions (schools open but stay-at-home orders in place; November 5, 2020-January 5, 2021, and March 9, 2021-April 30, 2021), and (3) strict restrictions (school closures and stay-at-home orders; January 6, 2021-March 8, 2021). Each week of the study was classified into one of these restriction categories so that the impact of the restriction levels on app engagement for each week of the study could be assessed.

### Statistical Analysis

Normally distributed variables were described as means and SDs, variables that were nonnormally distributed were described as medians and IQRs, and categorical variables were described as number and percentage. To test whether there were differences in baseline characteristics by participation in the app substudy (yes or no), Mann-Whitney 2-sample statistics were used for nonnormally distributed variables, 2-sample 2-tailed t tests were used for normally distributed variables, and chi-square tests were used for categorical variables.

Generalized estimated equation logistic regression models were used to assess whether engagement changed across the study period. An autoregressive covariance structure was used to allow for correlations to diminish over time, that is, to allow for measures of engagement for each COVID-19 digital biomarker module close in time to be more correlated than those further apart. Engagement categories for each study week were collapsed to 2 levels—no engagement (no module per week) and engagement (module completed ≥1 times per week)—and study time was divided into 3-monthly periods—1 to 13 weeks, 14 to 26 weeks, and ≥27 weeks—for ease of interpretation. Similar models were used to assess whether engagement differed between study weeks where symptoms were reported and weeks where symptoms were not reported and assess between weeks with different COVID-19 social restriction levels (minimal, moderate, and strict). Results from the models were expressed as odds ratios and 95% CIs.

To test whether there were differences in participant characteristics with continued engagement with the 3 digital biomarker modules, equality of medians tests were used for nonnormally distributed variables, fixed-effect ANOVAs were used for normally distributed variables, and chi-square tests were used for categorical variables.

Participant characteristics significantly associated with engagement level from these univariate models were entered into an ordered logistic regression model. Perceived stress scores were nonnormally distributed and categorized in quartiles. For ease of interpretation, age was categorized as 44 to <55 years, 55 to <65 years, and ≥65 years. Brant tests were used to test the proportional odds assumption.

Statistical analyses were performed using Stata (version 16.1; StataCorp) [25].

### Participant Interviews and Qualitative Data

Qualitative semistructured interviews were conducted with 35 participants: 22 (63%) who consented to the app substudy and 13 (37%) who did not consent to the app substudy but participated in the web-only study (participant recruitment information provided in Table S1 in Multimedia Appendix 2). Participants were purposively sampled to include a range of different ages, genders, educational attainment, and levels of engagement in the 3 digital biomarker modules (median 0, 1-2, or ≥3 times per week), with those recruited into the app substudy double sampled as they would provide rich experiences of using the app and taking part in the study (Table S2 in Multimedia Appendix 2). Participants were emailed invitations to participate in an interview.

Interviews were conducted after the app had closed to data collection via a web-based meeting in a conferencing platform or via telephone, depending on participant preference. Interviews used a flexible interview schedule that was created by centering on the aims of the study, author expertise and discussions, and revision by the Fenland PPI panel. After 3 interviews, the appropriateness of the interview schedule and initial data was discussed by ERL and KR (these interviews were included in the main analysis). Questions for those who consented to the app substudy focused on reasons for consenting, experiences of initial app engagement, experiences completing measurements and inputting data, and factors influencing continued app engagement. Questions for those who did not consent focused on their reasons for not participating. Interviews were conducted by an experienced qualitative postdoctoral researcher (ERL) who had no involvement in the conduct of the study and had not had any contact with the participants before the interview. In all interviews apart from one (partner to help with translation), no one else was present. The interviews lasted, on average, 29 (SD 9.4) minutes. The interviews were audio recorded, transcribed verbatim, and anonymized.

The interviews were analyzed using a deductive thematic approach [26] regarding the study aims and the interview schedule questions to provide a focused analysis. Initially, 9% (3/35) of the interviews were coded independently by 2 researchers (ERL and RF) and then discussed to ensure consistency and appropriateness of the coding framework before continuing analysis of the remaining interviews. Analysis was conducted by ERL, and 100% (35/35) of the interviews were double-checked by a second researcher (RF), both of whom met throughout the process to discuss and iteratively refine findings. Findings were also discussed with KR to gain additional insights. Data were managed using NVivo software (version 12; QSR International) [27]. An exploratory-sequential approach was used with qualitative data to interpret and explain the quantitative data.

### Ethics Approval

Ethics approval for the Fenland COVID-19 study was obtained from the Southwest Cornwall and Plymouth Research Ethics committee (20/SW/0100). The Fenland PPI panel was involved in planning, conducting, and reporting the Fenland COVID-19 study.

## Results

### Initial Engagement: Quantitative Results

Overall, 4031 participants aged 44 to 70 years consented to take part in the Fenland COVID-19 study. Of these 4031 participants, 2524 (62.61%) also consented to be in the app substudy. Baseline characteristics of both cohorts are outlined in Table 1. There were some differences between participants who consented to take part in the app substudy and those who consented to be in the main web-only study (Table 1).

Those who consented to the app substudy were more likely to be men, in the highest socioeconomic status category, and in employment and have a university degree than those who did not consent. They were also more likely to live in an urban area, less likely to live in an area categorized as being deprived, and more likely to live with family instead of with friends or partners or alone.

With regard to participants’ health status, there were no differences between the app substudy and the web-only study in smoking status or self-rated health. However, when compared with those who only participated in the web-only study, those in the app substudy had, on average, a slightly higher BMI and were less likely to report having any health conditions and poorer physical health. The prevalence of specific conditions also varied between the groups. For example, those in the app substudy were more likely to report having asthma and musculoskeletal conditions but less likely to report anxiety or depression compared with those who only participated in the web-only study.

Table 1 also examines whether having had a COVID-19 infection before the study (indicated by a positive antibody test result) influenced participants’ choice in joining the app substudy. There was no difference in the proportion of participants who had a positive antibody test at baseline. Furthermore, there were only 6 recorded positive COVID-19 antigen test results before recruitment (app substudy: n=3, 50%; web-only study: n=3, 50%).

Of those who consented to take part in the app substudy, 90.21% (2277/2524) completed the app onboarding process from August 2020 to October 2020 (downloading the app, app consent, and registering; Figure 1). Participants who completed the onboarding process used a large range of smartphone models (Table 2), with 53% (1207/2277) using an iOS device and 47% (1070/2277) using an Android device. The most popular iOS device used in the study was released in September 2016, with the oldest devices being from October 2014. For Android phones, operating systems give some indication of the age of the device or the regularity of the updates by the user; these ranged from 2015 to 2020.

**Table 1:** Baseline characteristics by consent status: app substudy or web-only (N=4031).

Characteristics				App substudy (n=2524)		Web-only (n=1507)		*P* value	
**Demographic**									
	Sex (male), n (%)		1149 (45.52)		614 (40.74)		.003		
	Age (years), mean (SD)		58.4 (7)		59.7 (7.1)		<.001		
	Ethnicity (White), n (%)		2405 (98.16)^a^		1419 (97.33)^b^		.08		
	**Living status, n (%)**								<.001
		Living alone	194 (8.47)^c^		153 (11.7)^d^				
		Living with family	1291 (56.38)^c^		658 (50.31)^d^				
		Living with friends or partner	802 (35.02)^c^		491 (37.54)^d^				
		Other	3 (0.13)^c^		6 (0.38)^d^				
**Socioeconomic, n (%)**									
	**SES^e^ category**								<.001
		Traditional and modern professional and higher managerial occupations	1654 (67.7)^f^		871 (59.94)^g^				
		Lower managerial and intermediate occupations	428 (17.52)^f^		310 (21.34)^g^				
		Technical or semiroutine and routine occupations	361 (14.78)^f^		272 (18.72)^g^				
	Currently working		1388 (65.41)^h^		658 (58.23)^i^		<.001		
	Higher degree		1194 (47.36)^j^		624 (41.46)^k^		<.001		
	Residence in urban area		1283 (51.05)^l^		753 (50.23)^m^		.04		
	Living in deprived area		285 (11.35)^n^		204 (13.64)^o^		.03		
**Health**									
	**Smoking status, n (%)**								.23
		Current smoker	82 (3.56)^p^		58 (4.34)^q^				
		Ex-smoker	835 (36.23)^p^		453 (33.93)^q^				
		Nonsmoker	1388 (60.22)^p^		824 (61.72)^q^				
	BMI (kg/m^2^), median (IQR)		25.8 (23.2-29.0)^r^		25.5 (22.8-28.7)^s^		.03		
	Physical activity energy expenditure (kJ/kg/day), median (IQR)		31.6 (20.3-46.9)^h^		31.2 (20.8-46.3)^i^		.88		
	**Self-rated health, n (%)**								.26
		Poor or fair	379 (16.44)^p^		236 (17.92)^t^				
		Good or excellent	1926 (83.56)^p^		1081 (82.08)^t^				
	**Number of self-reported health conditions, n (%)**								<.001
		0	1278 (55.44)^p^		694 (52.7)^u^				
		1	494 (21.43)^p^		391 (29.69)^u^				
		≥2	533 (23.12)^p^		232 (17.62)^u^				
	**Most prevalent self-reported health conditions, n (%)^v^**								
		High blood pressure	353 (15.31)^p^		218 (16.31)^w^		.43		
		Asthma	288 (12.49)^p^		120 (8.98)^w^		.001		
		Anxiety	135 (5.86)^p^		118 (8.96)^w^		<.001		
		Depression	138 (5.99)^p^		102 (7.74)^w^		.04		
		Musculoskeletal^x^	179 (7.77)^p^		12 (0.9)^w^		<.001		
	**SARS-CoV-2 IgG^y^ antibody status, n (%)**								.69
		Positive	149 (6.19)^z^		76 (5.86)^aa^				
		Negative	2257 (93.81)^z^		1220 (94.13)^aa^				
	**Physical function, n (%)**								.02
		Good	2272 (99.21)^c^		1287 (98.39)^d^				
		Poor to moderate	18 (0.79)^c^		21 (1.61)^d^				

^a^n=2450.

^b^n=1458.

^c^n=2290.

^d^n=1308.

^e^SES: socioeconomic status.

^f^n=2443.

^g^n=1453.

^h^n=2122.

^i^n=1130.

^j^n=2521.

^k^n=1505.

^l^n=2513.

^m^n=1499.

^n^n=2510.

^o^n=1495.

^p^n=2305.

^q^n=1335.

^r^n=2049.

^s^n=1241.

^t^n=1081.

^u^n=1317.

^v^Top 5 self-reported health conditions.

^w^n=1337.

^x^Diseases of the musculoskeletal system and connective tissue.

^y^IgG: immunoglobulin G.

^z^n=2406.

^aa^n=1296.

**Table 2:** Smartphone devices used in the app substudy by release year (N=2250).

Year of release	Users, n (%)
2020	394 (17.51)
2019	568 (25.24)
2018	446 (19.82)
2017	296 (13.16)
2016	378 (16.8)
2015	161 (7.16)
2014	7 (0.31)

### Retention

Participants who stopped taking part in the app substudy either withdrew from the study completely or decided to stop participating in the app substudy but remained participating in the Fenland COVID-19 study (web-only). Overall, 3.72% (150/4031) of participants withdrew from the Fenland COVID-19 study between consenting to take part and the end of the 6-month study period (Figure 1). A significantly higher proportion of these participants came from the web-only study compared with the app substudy (87/1507, 5.77% vs 63/2524, 2.5%, respectively; *P*<.001). In addition, 3.41% (86/2524) of the participants who consented into the app substudy chose to withdraw from the app substudy during the study period but remained participating in the web-only study.

### Initial Engagement: Qualitative Results

#### Reasons for Not Consenting to the App Substudy

In interviews with participants in the web-only study (no experience of using the app), a main reason reported for not consenting to the app substudy was not being aware of receiving the invitation. Participants noted that they also may not have properly read the invitation and, therefore, may have misunderstood that it was different from the web-only study:

No, we must’ve missed it somewhere along the lines. I don’t remember even seeing it...

Participant 22

I didn’t read it well enough clearly because I didn’t pick that bit up.

Participant 35

...I just thought I would be duplicating what I was already doing, so that was the reason why.

Participant 21

A few of these participants added that they would likely have taken part in the app substudy if they had known that they were invited and that it provided information additional to the survey:

Yeah, I would have gone “okay, so if that helps you more, absolutely.” Why wouldn’t I?

Participant 35

An additional reason for not participating in the app study included participants assuming that their mobile phone could not support the software as it was too old, lacked sufficient memory, or was not a “smart phone”; therefore, they disregarded or did not attempt to engage in the app substudy from the outset:

I hadn’t got a phone that was updated enough, so I disregarded that bit, because it didn’t really apply to me.

Participant 17

I had no memory to be able to upload anything onto it, so, yeah, I couldn’t do the app. I would have loved to have done the app actually, but I couldn’t do it.

Participant 33

A few participants also noted that they did not use or like mobile phones and apps:

I don’t tend to use a mobile. I’m one of those people that’s not a big mobile fan. My mobile’s mostly at home, I don’t take it out and about with me, so it’s not something that I use very often. So I’d rather just log onto a website than have an app on my phone.

Participant 31

#### Reasons for Consenting to the App Substudy

Most participants who consented to the app substudy reported taking part for altruistic reasons and that they wanted to “...contribute during the lockdown to something that’s good...” (Participant 24).

Some participants described the app substudy as being a good, sensible addition to the Fenland study. Having previous positive experiences of the Fenland study was also influential:

...the Fenland Study was organised so well, I thought...it’s a worthwhile work and, you know, the infrastructure is there to do this quite effectively.

Participant 2

Some of the participants reported that they were usually interested and keen to take part in health research. Contributing to the understanding of COVID-19 was also mentioned to be particularly motivating to participate in the study, and a small number were curious to find out their COVID-19 status.

#### Experience of the Initial Study Process

Interviewed participants who consented to the app study generally had a positive experience with the onboarding process, describing it as straightforward, smooth, easy, and relatively simple. Participants reported the instructions related to onboarding to be clear and comprehensive:

To be honest it was spot on. You know, it told you how to download the app, what to do, how to sign in, it told you everything that you needed to know.

Participant 1

However, some participants also could not remember if they used the instructions, and a participant reported wanting more information on locating and downloading the app.

### Continued Engagement: Quantitative Results

The study period for all participants was a minimum of 6 months (28 weeks) and could be longer depending on the date they started the study. The median time in the study was 34.5 weeks (IQR 34-37), with a total of 77,893 weeks across all the participants. During the study period, a median of 277 (IQR 80-374) separate module entries were completed per participant (RHR, temperature, oxygen saturation, and symptom modules). The number of modules completed across the study period was very similar between the COVID-19 digital biomarker modules; the RHR module was completed at least once in 72.77% (56,517/77,664) of the study weeks, body temperature was completed in 73.06% (56,742/77,664) of the study weeks, and oxygen saturation was completed in 73.51% (57,088/77,664) of the study weeks. Completion of the self-reporting symptom module was substantially lower, with 48.71% (37,834/77,664) of the study weeks having one or more entries.

**Figure 4:**
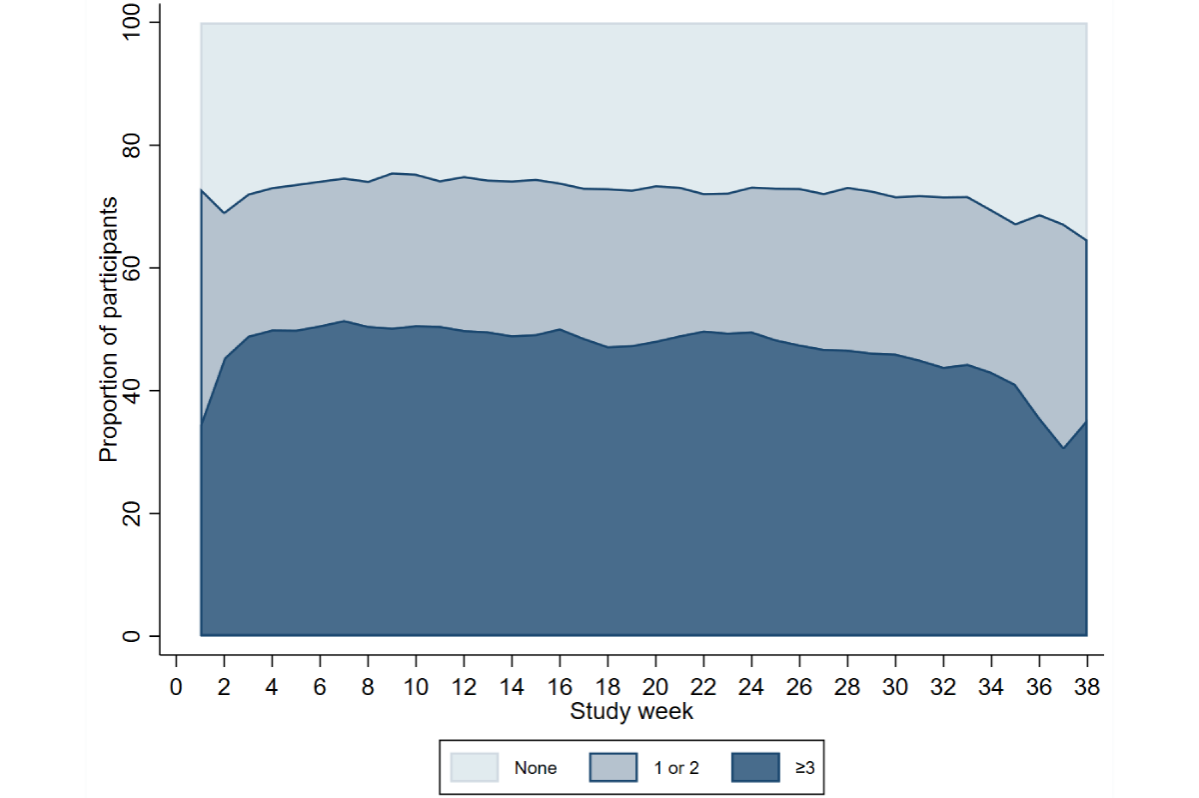
Completion frequency categories of weekly resting heart rate modules across study weeks.

**Figure 5:**
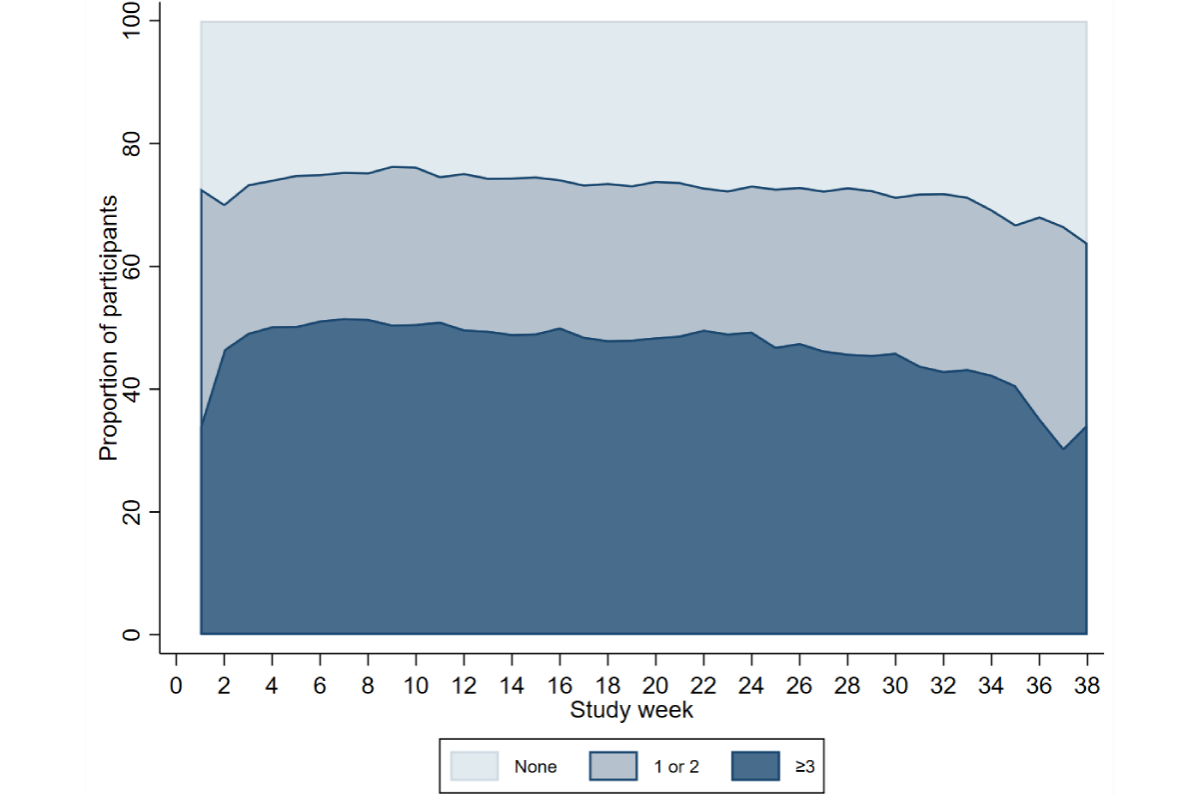
Completion frequency categories of weekly body temperature modules across study weeks.

**Figure 6:**
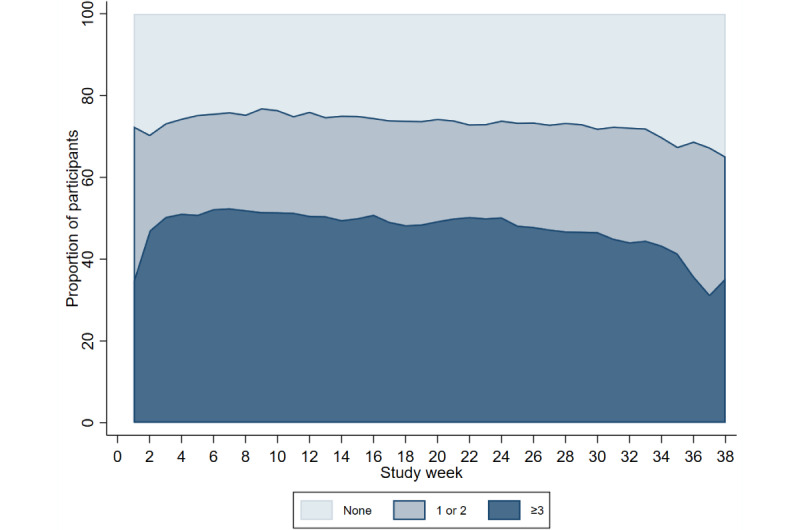
Completion frequency categories of weekly oxygen saturation modules across study weeks.

**Figure 7:**
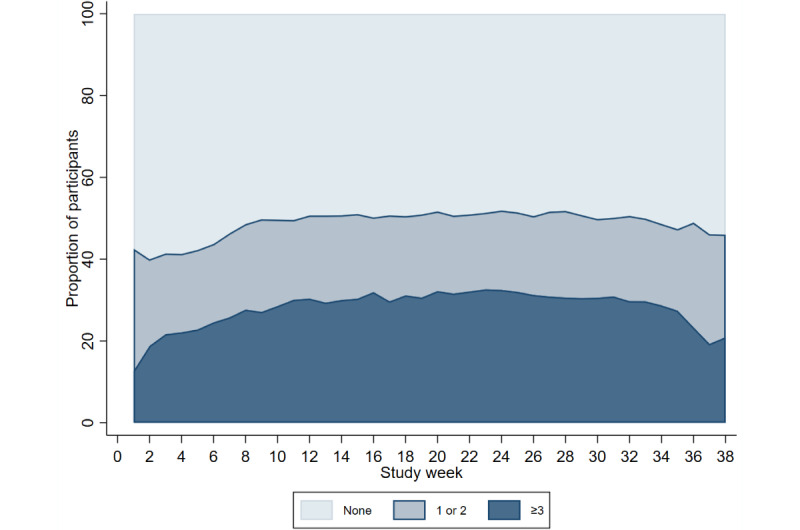
Completion frequency categories of weekly symptoms module across study weeks.

Completion of the modules remained constant across the study period (Figures 4-7). Population-averaged effects were examined to assess engagement for the COVID-19 digital biomarker modules across different phases of the study period (0-3 months, 3-6 months, and ≥6 months). There was no change in engagement with any of the digital biomarker modules during 0 to 3 months or 3 to 6 months of the study period (Table 3). The reduction in engagement came in the last study period beyond 6 months, where there was a significant reduction in engagement in all 3 COVID-19 digital biomarker modules.

**Table 3:** Population-averaged effects of engagement with COVID-19 digital biomarker modules by study period by weeks with reported symptoms and differing COVID-19 social restriction levels.

			Total study week observations, N		Digital biomarker module, OR^a^ (95% CI)				
					Resting heart rate module		Body temperature module		Oxygen saturation module
**Study period (weeks)**									
	1 to 13	28,981		1.01 (0.99-1.02)		1.00 (0.99-1.02)		1.01 (0.99-1.02)	
	14 to 26	28,542		0.99 (0.98-1.00)		0.99 (0.98-1.00)		0.99 (0.98-1.00)	
	≥27	20,141		0.95 (0.94-0.97)		0.95 (0.94-0.96)		0.95 (0.94-0.96)	
**Reported symptoms^b^**									
	No	39,830		Reference		Reference		Reference	
	Yes	37,834		1.59 (0.97-2.60)		1.29 (0.69-2.38)		2.50 (1.20-5.22)	
**Restriction category^c^**									
	Minimal	N/A^d^		Reference		Reference		Reference	
	Moderate	N/A		1.03 (0.97-1.09)		1.04 (0.98-1.10)		1.04 (0.98-1.11)	
	Strict	N/A		1.06 (0.99-1.14)		1.08 (1.01-1.16)		1.08 (1.01-1.16)	

^a^OR: odds ratio.

^b^Adjusted for study period.

^c^Restriction categories: minimal restrictions (August 6, 2020, to November 4, 2020), moderate restrictions (November 5, 2020, to January 5, 2021, and March 9, 2021, to April 30, 2021), and strict restrictions (January 6, 2021, to March 8, 2021) adjusted for calendar weeks.

^d^N/A: not applicable.

### Continued Engagement: Qualitative Results

#### Ease of Using the App

Overall, it was reported by the participants who consented to the app substudy that the app was easy to navigate and self-explanatory on how to input the data. Participants suggested that, in general, inputting measures into the app caused none or few problems:

...I remember it was just step-by-step, press this or press this. It was very easy.

Participant 28

...it seemed to work very straightforwardly for me. I can’t remember having a difficulty with it...

Participant 32

Instructions provided to support participants on taking measures and inputting their data into the app were generally well received, although a very small number of participants stated that the instructions were too long, some sections were not clear, or they did not recall using them.

Most participants also found the frequency of times per week and length of time “manageable”:

Didn’t take long, that’s what I like about anything, if it doesn’t take up too much of my time!

Participant 6

#### Experiences Using the Equipment

Most participants reported that they liked measures (eg, RHR) that were built into the app rather than requiring additional devices:

Oh, I loved the heart rate monitor...because it was inbuilt...because it was not an additional bit of equipment to use.

Participant 10

A few reported the additional devices to be inconvenient when traveling (eg, holidays or traveling for work) and they could be left behind.

A small number of participants reported malfunctioning equipment that prevented them from inputting data for temperature and oxygen saturation. However, most participants reported having no issues with the equipment:

...the thermometer at one point died so I just bought a new thermometer and used my own...

Participant 18

...the pulse oximeter stopped working but that wasn’t a problem, I contacted them [study team] and they sent another one straight out.

Participant 18

Participants noted some occasions when they had difficulty obtaining a correct reading for RHR and temperature. For example, a participant used the wrong part of the phone to measure their RHR. A small number of participants disliked using the thermometer and sometimes obtained a low temperature reading that was outside the accepted range to enter into the app, so the app did not allow them to input it:

...if you’re not that familiar with it then you might’ve got confused...I think I put my finger on the camera operating button or something daft like that.

Participant 19

...at times was awkward, if I didn’t quite get it in the right place in my tongue I’d end up with a low figure, which obviously wasn’t correct so I’d start it all over again.

Participant 4

#### Self-reported Symptoms

For symptom reporting, most participants reported having minimal issues entering the data, predominantly as they did not have symptoms. However, some participants also reported not entering data as they did not think it was necessary if they were symptom-free:

...I didn’t have symptoms all the way through so I think I just ignored it because there wasn’t a question of have you not had symptoms for the last month or whatever.

Participant 13

...because I had no symptoms I just left it blank, I didn’t think I had to write, “no symptoms,” in it and that’s just, that’s just user error.

Participant 18

#### App Malfunction

A small number of participants highlighted very occasional app malfunctions or connection problems (eg, lack of Wi-Fi). These issues were usually resolved by the Huma app help desk or by sending instructions to the participants. On a few occasions, participants resolved the issues by simply trying again later:

...something sort of went wrong and we got a message saying they were working on it. And it got sorted in a day or so, I seem to think.

Participant 11

...they sort of like texted me saying that I’ve got to re-log, login because there’s a problem with the app.

Participant 16

However, a small number of participants were unable to resolve software issues, with reports that it did not work reliably or the app had to be reinstalled every time data were to be entered.

### Factors and Participant Characteristics Associated With Continued Engagement: Quantitative Results

The effect of experiencing symptoms on engagement with COVID-19 digital biomarker modules was also assessed. Level of engagement was compared between weeks where participants had reported possible COVID-19 symptoms and weeks where no symptoms were reported (either entered as “no symptoms” in the app or no entries made). Overall, 56% (1310/2339) of participants reported experiencing symptoms at least once over the study period. Symptoms were reported in 29.41% (11,126/37,834) of the study weeks versus no symptoms reported in 70.59% (26,708/37,834) of the study weeks. Engagement in the oxygen saturation module was significantly higher in weeks where participants reported symptoms, but symptom reporting did not relate to engagement with the RHR or temperature modules.

The effect of the national COVID-19 social restriction periods (categories: minimal, moderate, or severe) on engagement was compared adjusting for calendar weeks. The proportions of measurements in each restriction category were very similar between the COVID-19 digital biomarker modules (Figure S1 in Multimedia Appendix 3). No differences were observed in engagement between the different restriction periods for the RHR module. During the weeks of the most severe restrictions, engagement in the temperature and oxygen saturation modules was slightly higher compared with the minimal restriction period.

Owing to the consistency in engagement between the 3 COVID-19 digital biomarker modules, we combined the data from each digital biomarker to give an overall median level of engagement category—no interaction, 1 to 2 times per week, and ≥3 times per week—which we used to assess what factors were associated with higher engagement. Participants had been asked to complete these modules 3 times per week, so the highest category of engagement (median ≥3 times per week) corresponded to consistently meeting this maximum engagement level across the study period.

Higher engagement was associated with being older, not working, and living alone or with friends or partners than with living with family (Table 4). There was no difference in the proportion of men and women; of education level; or of those living in an urban, rural, or deprived area between the engagement categories. A higher proportion of participants from lower managerial and intermediate occupations were in the highest category of engagement than in the lower engagement categories, but there were no differences in the other indicators of socioeconomic status between the engagement categories. In terms of health factors, those who engaged the most with the digital biomarker modules had a lower BMI, were less likely to be current smokers, were less likely to be depressed, and had a lower perceived stress score than those who engaged less with the modules. However, there were no differences in engagement related to self-rated health, physical function, generalized anxiety disorder, or reported health conditions. Having had a positive COVID-19 antigen test result or a positive antibody test result during the study was not associated with level of engagement. In addition, there were no differences in the proportion of participants having a positive antigen test during the study between those who consented to take part in the app substudy and those who did not (80/524, 15.3% vs 47/344, 13.7%, respectively; *P*=.50), and the proportion of participants who had at least one positive antibody test result during the study period was also similar (app substudy: 321/2438, 13.17% vs web-only: 188/1339, 14.04%).

Those participant characteristics significantly associated with engagement level from the univariate models were entered into an ordered logistic regression model (Table 5). Older age groups and lower managerial and intermediate occupations remained significantly associated with higher engagement in the COVID-19 digital biomarker modules, whereas working, being a current smoker, and being overweight or obese were associated with lower engagement. The highest perceived stress score quartile was associated with lower engagement, but no association was observed with the lower perceived stress score quartiles or with depression (Patient Health Questionnaire-8).

**Table 4:** Participant characteristics associated with level of engagement with COVID-19 digital biomarker modules.

				Median level of engagement with app modules							*P* value
				0 (no interaction)		1 (1-2 times per week)		2 (≥3 times per week)			
Participants, n (%)				568 (25.2)		575 (25.6)		1107 (49.2)		N/A^a^	
**Demographic characteristics**											
	Sex (male), n (%)		269 (47.4)		264 (45.9)		492 (44.4)		.52		
	Age (years), mean (SD)		56.5 (6.7)		57.2 (7.1)		59.8 (6.8)		<.001		
	**Living status, n (%)**									<.001	
		Living alone	46 (8.1)		43 (7.5)		98 (8.9)				
		Living with family	360 (63.6)		345 (60.5)		566 (50.5)				
		Living with friends or partner	160 (28.2)		181 (31.8)		433 (39.4)				
		Other	0 (0)		1 (0.2)		2 (0.2)				
**Socioeconomic characteristics, n (%)**											
	**SES^b^ category**									.01	
		Traditional and modern professional and higher managerial occupations	369 (67.3)		411 (72.9)		700 (65.8)				
		Lower managerial and intermediate occupations	88 (16.1)		79 (14)		211 (19.8)				
		Technical or semiroutine and routine occupations	91 (16.6)		74 (13.1)		153 (14.4)				
	Currently working		327 (75)		348 (70.5)		605 (58.7)		<.001		
	Higher degree		257 (45.3)		278 (48.4)		539 (48.8)		.37		
	Residence in urban area		284 (50.3)		283 (49.5)		563 (51)		.56		
	Living in deprived area		66 (11.7)		67 (11.7)		111 (10.1)		.48		
**Health characteristics**											
	Smoking status—current smoker, n (%)		31 (5.5)		28 (4.9)		23 (2.1)		<.001		
	BMI (kg/m^2^), median (IQR)		26.8 (24.1-30.5)		26.2 (23.8-29.3)		25.4 (22.8-28.4)		<.001		
	Physical activity energy expenditure (kJ/kg/day), median (IQR)		32.7 (19.8-47.0)		31.5 (21.4-47.6)		31.6 (20.4-45.8)		.86		
	**Mental health measures**										
		Generalized anxiety disorder (GAD^c^ ≥10), n (%)	20 (5.8)		30 (5.3)		44 (4)		.28		
		Perceived stress score, median (IQR)	13 (7-18)		11 (6-16)		11 (6-15)		<.001		
		Depression (PHQ-8^d^ ≥10), n (%)	38 (11.2)		35 (6.2)		59 (5.4)		<.001		
	**Self-rated health, n (%)**									.28	
		Poor or fair	105 (18.5)		95 (16.5)		171 (15.5)				
		Good or excellent	463 (81.5)		480 (83.5)		936 (84.5)				
	**Number of self-reported health conditions, n (%)**									.91	
		0	306 (53.9)		314 (54.6)		623 (56.2)				
		1	126 (22.2)		125 (21.7)		231 (20.9)				
		≥2	136 (23.9)		136 (23.7)		253 (22.9)				
	**Most prevalent health conditions, n (%)^e^**										
		High blood pressure	94 (16.6)		102 (17.7)		150 (13.6)		.05		
		Asthma	79 (13.9)		74 (12.9)		129 (11.7)		.40		
		Anxiety	42 (7.4)		29 (5)		57 (5.2)		.13		
		Depression	44 (7.8)		31 (5.4)		60 (5.4)		.13		
		Musculoskeletal^f^	46 (8.1)		46 (8)		87 (7.9)		.98		
	Positive SARS-CoV-2 IgG^g^ antibody status during the study, n (%)		75 (13.8)		66 (11.5)		144 (13.1)		.50		
	**Recorded COVID-19 antigen test result during study period, n (%)**									.76	
		Positive	23 (14.8)		21 (17.8)		29 (15)				
		Negative	132 (85.2)		97 (82.2)		164 (85)				
	**Physical function, n (%)**									.94	
		Good	561 (99.1)		566 (99.3)		1090 (99.2)				
		Poor to moderate	5 (0.9)		4 (0.7)		9 (0.8)				

^a^N/A: not applicable.

^b^SES: socioeconomic status.

^c^GAD: Generalized Anxiety Disorder scale.

^d^PHQ-8: Patient Health Questionnaire-8.

^e^Top 5 self-reported health conditions.

^f^Diseases of the musculoskeletal system and connective tissue.

^g^IgG: immunoglobulin G.

**Table 5:** Ordered logistic regression model of participant characteristics associated with level of engagement with COVID-19 digital biomarker modules.

Independent variable and category		OR^a^ (95% CI)
**Age category (years)**		
	44 to <55	Reference
	55 to <65	1.62 (1.28-2.04)
	≥65	2.14 (1.55-2.94)
**Working status**		
	Not working	Reference
	Working	0.71 (0.56-0.89)
**Living status**		
	Living alone	Reference
	Living with family	0.70 (0.48-1.04)
	Living with friends or partner	1.0 (0.66-1.50)
	Other	1.08 (0.97-12.0)
**SES^b^ category**		
	Traditional and modern professional and higher managerial occupations	Reference
	Lower managerial and intermediate occupations	1.37 (1.04-1.80)
	Technical or semiroutine and routine occupations	1.21 (0.89-1.64)
**Smoking status**		
	Not smoking	Reference
	Current smoker	0.56 (0.32-0.99)
**BMI category (kg/m^2^)**		
	Healthy weight	Reference
	Overweight	0.72 (0.58-0.90)
	Obese	0.59 (0.45-0.79)
**Perceived stress score quartiles**		
	Lowest	Reference
	Second	0.92 (0.69-1.24)
	Third	1.08 (0.80-1.46)
	Highest	0.69 (0.51-0.94)
**Depression (PHQ-8^c^)**		
	<10	Reference
	≥10	0.84 (0.53-1.34)

^a^OR: odds ratio.

^b^SES: socioeconomic status.

^c^PHQ-8: Patient Health Questionnaire-8.

### Factors and Participant Characteristics Associated With Continued Engagement: Qualitative Results

#### Establishing a Routine

Participants reported that building the measurements into their daily routine supported data input. Reasons for not completing the measurements were commonly because of a change in routine (eg, early work shift or run) and commitments or priorities (eg, new grandchild, childcare arrangements, or family illness):

Doing the readings wasn’t a problem and if I had a normal routine it would be fairly easy to fit in with, but because of the Covid-19 situation and the age of our grandchildren...our routine is a bit sort of messed around...sometimes you forget but obviously otherwise it wouldn’t be a problem.

Participant 19

A small number of participants noted that trying to establish a regular routine was challenging, with difficulties including trying to spread the measures evenly across the week and needing self-discipline. Some felt that they simply forgot or needed to find time if they were busy:

...just remembering to do it, particularly if you knew you’d got a very busy day, that’s all really...The day-to-day inputting of like your temperature, pulse, whatever wasn’t a hassle at all.

Participant 18

#### Ability to Monitor Their Own Health

Some participants found measuring body temperature and oxygen saturation to be useful for monitoring signs of COVID-19 and provided a sense of security:

...there was a sort of comfort in that...you were sort of testing yourself regularly as an early indication if something else had happened...

Participant 12

...I suppose it gave me a bit of an added security making sure my temperature was okay every day...

Participant 18

#### Effect of Social Restriction Periods and COVID-19 Infection Rates

Some participants felt that national COVID-19 social restriction periods did not affect the level of data input in comparison with times without restrictions. This was related to working and cohabitation status as many were retired or living alone and, therefore, perceived social restrictions had little impact on their daily lives and activities:

Well, as I’m retired, to be honest there wasn’t a lot of difference in my daily routine, I’d say.

Participant 10

Those working also generally felt that social restriction periods had little influence on engagement levels:

No I’d be, I’d be fine any time really, because I wasn’t off during the pandemic at all, I worked all the way through it, yeah.

Participant 29

A participant reported that there were dips in their participation when COVID-19 rates were low:

There was a period I suppose between waves of COVID when there was a feeling of “is this still helpful, is this still necessary?” then the second wave hit which was absolutely convincing, yes of course it is.

Participant 12

The same participant also stated that the study was beneficial in giving them a routine during the lockdown:

Well, just the regularity of taking my temperature and get my blood oxygen level, so at a time when everything else seemed to disappear, sort of sports and all the other reasons that get me out of bed in the morning that it was good to have a replacement and the study sort of, you know, did take that role in some respects.

Participant 12

#### Age

A small number of participants acknowledged that increasing age may influence app engagement, perceiving that those of an older age may not own or be less familiar with apps and mobile phones. However, a participant suggested that social distancing restrictions had possibly resulted in this group becoming more accustomed to using this technology:

...I’m almost 50 so I have this gadget, it takes me a little bit longer to get them but I got used to them...So that’s a bit of a limit but doesn’t exclude you from doing these studies and nowadays with all the lockdowns and whatever I think the population is digitally more educated anyway, so they found out things in lockdown.

Participant 5

#### Waning Interest During the Study Period

A small number of participants reported times that they felt their data input had reduced because of waning interest in the app or not knowing if information was still required. A few also reported that they felt that it became boring and repetitive because of registering the same information every day, although notifications from the study and being so close to the end encouraged them to continue:

...I also had assumed that, you know, information wasn’t required. But then I think there was an email to say make sure that we give information right up to the end of the study.

Participant 26

...I got the notification that it was finishing anyway, so it just seemed petty to come out of it for a fortnight.

Participant 13

#### Wish to Complete the Study

Most participants reported that they wanted to continue to the end of the study as they felt that there was no point in stopping early. In addition, participants reported continuing to ensure that the information collected was useful for the study. Most of the interviewed participants reported that they continued in the study until they were told to stop, and some would have continued for a longer period if required:

I can’t see the point in doing it halfheartedly, if you’re going to do it, you do it to the end.

Participant 29

Not for a specific length of time, just as long as I felt I was being useful and would’ve carried on with the app and the study if it had continued, I would’ve kept going.

Participant 12

#### Engagement Beyond the Pandemic

When asked if their engagement in the app substudy was driven by COVID-19 and the pandemic restrictions and if they would have engaged to the same level if it were for a different health outcome, participants felt that they would have. A small number of participants stated that they would take part again in an app study for other health conditions but depending on how important they thought the aim of the study was:

I, for the amount of time it took up, I’d quite happily do it at any time.

Participant 11

...I’m interested in health and keeping sort of healthy, so I would do it anyway I think, no problem.

Participant 9

## Discussion

### Principal Findings

This population-based study with >2000 participants assessed what factors were associated with both initial and continued engagement with an mHealth platform. This mixed methods study demonstrated both high initial and sustained engagement over the 6-month period of the study with active remote measurements, and the qualitative results provided valuable insights from the user perspective. Over the study period, withdrawal from the app substudy was low (64/2524, 2.54%), and completion of the modules remained consistently high. Enrollment and retention in traditional cohort-based epidemiological studies is a constant challenge, with participation declining in recent years [28].

Active digital measurements in this study were considerably more frequent (3 times per week) and lasted for a longer period (minimum of 6 months) than in many other published studies that have assessed engagement with mHealth digital measures [8,29]. Using mHealth platforms allows for very frequent data collection on a range of health behaviors, symptoms, and digital biomarkers. Such data collection is in sharp contrast to the more traditional cohort designs, with often long intervals between measurements with participants having to travel to a research facility to complete measurements. Collecting frequent real-world data from mHealth systems allows for better characterization of exposures and intermediate outcomes (such as biomarkers) and the ability to assess the impact of changes and within-person fluctuations over time [29]. However, the importance of clearly communicating the ongoing need for frequent data collection and the length of the study is important for engagement, as shown in the qualitative results of this study. Most studies assessing users’ adherence and engagement with mHealth systems have been clinical studies, often in specific therapeutic areas requiring condition-specific configurations [30]. Population studies using mHealth often look at specific groups such as athletes [31], college students [32], or health care workers [33]. Some larger studies have been conducted but for a specific purpose such as validation of the remote monitoring technology [34] and particular wearable devices [35] or evaluating the impact of financial incentives [36].

### Fully Remote App Setup

In this study, the app setup and the measurements were fully remote such that there was no face-to-face interaction to either recruit participants to the study or set up the app or the measurements or during the study to aid engagement. Owing to the social restrictions in place during this study, we spent considerable effort to provide comprehensive instructions and simplify the steps for the participant to set up. Most who consented to take part in the study (2277/2524, 90.21%) downloaded the app and commenced logging measurements in it, and qualitative results showed that participants found this process overall clear and complete. This proportion was higher than reported in a randomized mHealth trial embedded in the Framingham Heart Study, where 202 participants were randomized to remote support (email and phone) or in-person support [37]. They found that initial setup and use of devices was substantially higher in the in-person arm compared with the remote arm (84%-99% across devices vs 41%-75% in the remote arm). However, after the initial engagement, connected device use was similar in the trial over the 5-month study period between the 2 study arms, supporting the importance of the initial engagement.

### Nested App Studies

A strength of this study over population-based citizen science studies [29,38,39] is that participants were recruited from an established and well-characterized cohort, allowing for investigation of differences in sociodemographic and health characteristics of those who consented and engaged and those who did not. A nested study design allowed for the identification of sociodemographic differences and potential participant bias; participants were more likely to be men and slightly younger, come from higher–socioeconomic-status groups, and be more educated. However, it is important that this selection bias has been reported for all types of epidemiological studies and is not unique to mHealth research [40]. There are some limitations to this study. We did not collect information from all participants on their reasons for not taking part in the app substudy or reasons for withdrawing from the study. However, we did purposively sample in the qualitative study those who did not consent to the app substudy to investigate the reasons for not participating. The Fenland cohort study was recruited from Cambridgeshire in the United Kingdom, which geographically has a relatively low ethnic diversity and, although some areas of deprivation are apparent, it does not include very deprived areas, particularly urban deprivation. Therefore, some of the results may not be generalizable to other settings.

Overall, we found some differences in self-reported health, with those who consented to the app substudy more likely to report 2 or more health conditions and report having asthma and musculoskeletal conditions than those in the web-only study, but there was no difference in self-rated health, smoking status, or physical activity. However, those in the web-only study were more likely to report having anxiety or depression and poor to moderate physical function.

### Broadening Inclusivity in Digital Research

Broadening inclusivity in digital research is a major consideration for population-based research. Participants in this study were middle-aged and older adults (aged 45-70 years), which limits the generalizability of the results of this study to other age groups, which may face different engagement issues. However, this age group is important in population research, particularly for chronic diseases. Older age groups may face more technology literacy issues than younger adults, who have grown up with smartphones and have the broadest use of technology. However, digital engagement has increased substantially in those aged ≥60 years in recent years, particularly during the COVID-19 pandemic [41], as identified by participants’ experiences in this study. To maximize inclusivity for those less familiar with using apps, we provided both written and video instructions on how to download and open the app and undertake the measurements and provided both a web-based and telephone help center for those who were experiencing problems or had questions. Participants reported that these instructions were clear and comprehensive, although a small number felt that they were too lengthy. Issues were also usually quickly resolved by contacting the help center. Other studies have reported that digital literacy and other barriers can be overcome through offering instructions and support from a study team in person or via a telephone call [42,43].

Many studies have been conducted on the iOS platform only, limiting the generalizability of engagement results [8,9,29]. The app used in this study was designed for use on both iOS and Android devices. We found that there was almost equal use of iOS and Android devices in the population (1207/2277, 53% using an iOS device and 1070/2277, 47% using an Android device) and a large range of devices and operating systems used, often several years old. This demonstrates the importance of ensuring that the platform is compatible with a range of devices.

We also decided not to use Bluetooth-enabled external devices for the temperature and oxygen saturation measurement modules because of our concern about technology literacy in this age group. This is also a consideration in studies in low- and middle-income countries where Bluetooth-connected devices may be less feasible. This meant that data collection in this study was more active than passive.

### Passive Versus Active Data Collection

Qualitative research has identified that passive data collection may facilitate good engagement [30], and many studies have reported waning active data collection with devices over a 3-month period [8]. Further research is required on the impact of the number of external devices and their interaction with the app on levels of engagement.

In this study, participants did not receive notifications to complete the digital biomarker modules but could set their own reminders if they wished. In the qualitative findings, few participants reported that they set these reminders in the app but, rather, they had their own cues and “routine” for completing the measurements. This finding is consistent with a systematic review that reported that notifications become less important once the measurements become part of the participants’ daily routine [7]. Qualitative findings have identified that user engagement is higher with passive data collection than modules that require active data collection [30], whereas barriers often include lack of available time [44]. We did find that those who were most engaged longer-term in the digital biomarker modules were more likely to be older and not working and less likely to live in a family environment, a sociodemographic group where it might be easier to keep to a routine and have more time to complete the measurements regularly.

### Participant Motivation Factors

This study suggests that individual-level factors may have had a larger influence on longer-term engagement than extrinsic factors such as national social restriction periods, which had minimal impact on engagement. Interestingly, the rate of completion of the digital biomarker modules was very similar, although some measurements required active data collection, use of devices, and manual entry of values. However, completion of the symptom module was noticeably lower. The qualitative data suggest that participants did not see the importance of reporting the absence of symptoms. Adherence to logging symptoms has (to date) only been reported in studies of patients with active chronic diseases rather than in healthy populations [45-47], and the engagement of symptom reporting has been associated with the perceived utility for the individual, and disengagement was noted when there was no sign of change [42]. In those weeks where participants reported symptoms, engagement with the oxygen saturation module was higher but not with the other digital biomarker modules. This could be due to the information in the public domain at the time regarding the use of pulse oximeters to monitor oxygen saturation at home if a person suspected that they had a COVID-19 infection, particularly identifying if they were becoming seriously unwell and required medical support [48,49].

The Fenland COVID-19 substudy was conducted to understand the presymptomatic to symptomatic stages of COVID-19 infection. Initial engagement in this study relating to COVID-19 was not related to having had a COVID-19 infection before the start of the study, and continued engagement with the app was also not related to having had a COVID-19 infection during the study. During such an unprecedented time as a global pandemic, participants may have been more motivated to take part and stay engaged with the measurements than in other health research. However, the qualitative findings suggest that participants would take part in such a study for other health conditions if they felt it was important. However, we did not collect the reasons for withdrawals or for not taking part in the study. From the qualitative work, we did identify that some participants did not realize that the second invitation they received was to take part in the app substudy and thought it was a repeat of the study (web-based only) that they had already consented to participate in.

From this mixed methods study, we have developed a checklist of recommendations on how to conduct successful population-based mHealth studies (Textbox 1).

There are emerging guidelines on how to monitor and evaluate digital health care interventions and deploying mHealth apps to health research [50,51]. However, the focus tends to be on the efficacy of an app and there are considerable gaps in terms of understanding participant engagement and adherence, particularly in population-based studies and using both quantitative and qualitative data [7,52]. This mixed methods study demonstrated both high initial and sustained engagement in an mHealth COVID-19 study over a ≥6-month period in a large study of middle-aged and older adults. Being nested in a known cohort study enabled the identification of participant characteristics and factors associated with both initial and long-term engagement for future applications in population-based health research.

**Checklist for mobile health (mHealth) engagement in population-based studies.**

**Checklist for mHealth engagement**

- Ensure that the remote measurement technology platform is compatible with both iOS and Android and with devices at least 5 years old
- Make the initial engagement as simple as possible with minimum steps to complete
- To reduce digital exclusion, produce both written and video instructions on how to download the app and complete the onboarding process and test these with users
- Be clear on the participant burden, communicating the length of the study and level of engagement required
- If feasible for the study design, establish a routine for active data collection, for example, specific days of the week
- Avoid active data questions where the default or most common answer is an absence or null response (eg, reporting no symptoms)
- Consider passive data collection options where appropriate
- Importance of collecting both quantitative and qualitative data to monitor engagement

## Appendix

Multimedia Appendix 1 Methods.

Multimedia Appendix 2 Supplementary tables.

Multimedia Appendix 3 Completion frequency categories of weekly modules by restriction category.

## Abbreviations

IgG: immunoglobulin G
mHealth: mobile health
PPI: Participant and Public Involvement
RHR: resting heart rate

## References

[1] (2021). Global strategy on digital health 2020-2025. World Health Organization.

[2] Blandford A, Wesson J, Amalberti R, AlHazme R, Allwihan R (2020). Opportunities and challenges for telehealth within, and beyond, a pandemic. Lancet Glob Health.

[3] NHS Long Term Plan. National Health Service.

[4] Davis MM, Freeman M, Kaye J, Vuckovic N, Buckley DI (2014). A systematic review of clinician and staff views on the acceptability of incorporating remote monitoring technology into primary care. Telemed J E Health.

[5] Vindrola-Padros C, Singh KE, Sidhu MS, Georghiou T, Sherlaw-Johnson C, Tomini SM, Inada-Kim M, Kirkham K, Streetly A, Cohen N, Fulop NJ (2021). Remote home monitoring (virtual wards) for confirmed or suspected COVID-19 patients: a rapid systematic review. EClinicalMedicine.

[6] Xue JZ, Smietana K, Poda P, Webster K, Yang G, Agrawal G (2020). Clinical trial recovery from COVID-19 disruption. Nat Rev Drug Discov.

[7] Simblett S, Greer B, Matcham F, Curtis H, Polhemus A, Ferrão J, Gamble P, Wykes T (2018). Barriers to and facilitators of engagement with remote measurement technology for managing health: systematic review and content analysis of findings. J Med Internet Res.

[8] McManus DD, Trinquart L, Benjamin EJ, Manders ES, Fusco K, Jung LS, Spartano NL, Kheterpal V, Nowak C, Sardana M, Murabito JM (2019). Design and preliminary findings from a new electronic cohort embedded in the Framingham heart study. J Med Internet Res.

[9] Radin JM, Steinhubl SR, Su AI, Bhargava H, Greenberg B, Bot BM, Doerr M, Topol EJ (2018). The Healthy Pregnancy Research Program: transforming pregnancy research through a ResearchKit app. NPJ Digit Med.

[10] Fenland Study - MRC Epidemiology Unit. University of Cambridge.

[11] Koulman A, Rennie KL, Parkington D, Tyrrell CS, Catt M, Gkrania-Klotsas E, Wareham NJ (2022). The development, validation and application of remote blood sample collection in telehealth programmes. J Telemed Telecare (forthcoming).

[12] Ainsworth BE, Haskell WL, Herrmann SD, Meckes N, Bassett Jr DR, Tudor-Locke C, Greer JL, Vezina J, Whitt-Glover MC, Leon AS (2011). 2011 Compendium of Physical Activities: a second update of codes and MET values. Med Sci Sports Exerc.

[13] Spitzer RL, Kroenke K, Williams JB, Löwe B (2006). A brief measure for assessing generalized anxiety disorder: the GAD-7. Arch Intern Med.

[14] Kroenke K, Spitzer RL, Williams JB (2001). The PHQ-9: validity of a brief depression severity measure. J Gen Intern Med.

[15] Kroenke K, Strine TW, Spitzer RL, Williams JB, Berry JT, Mokdad AH (2009). The PHQ-8 as a measure of current depression in the general population. J Affect Disord.

[16] Cohen S, Kamarck T, Mermelstein R (1983). A global measure of perceived stress. J Health Soc Behav.

[17] Information for Researchers - MRC Epidemiology Unit. University of Cambridge.

[18] Huma.

[19] Mol D, Riezebos RK, Marquering HA, Werner ME, Lobban TC, de Jong JS, de Groot JR (2020). Performance of an automated photoplethysmography-based artificial intelligence algorithm to detect atrial fibrillation. Cardiovasc Digit Health J.

[20] (2014). 2011 Local Authority Rural Urban Classification. United Kingdom Government.

[21] (2019). English indices of deprivation 2019: mapping resources. United Kingdom Government.

[22] Barrett P, Imamura F, Brage S, Griffin SJ, Wareham NJ, Forouhi NG (2017). Sociodemographic, lifestyle and behavioural factors associated with consumption of sweetened beverages among adults in Cambridgeshire, UK: the Fenland Study. Public Health Nutr.

[23] SOC2010 volume 3: the National Statistics Socio-economic classification (NS-SEC rebased on SOC2010). Office for National Statistics.

[24] Golubic R, May AM, Benjaminsen Borch K, Overvad K, Charles MA, Diaz MJ, Amiano P, Palli D, Valanou E, Vigl M, Franks PW, Wareham N, Ekelund U, Brage S (2014). Validity of electronically administered Recent Physical Activity Questionnaire (RPAQ) in ten European countries. PLoS One.

[25] (2019). Stata Statistical Software: Release 16.1. StataCorp.

[26] Braun V, Clarke V (2006). Using thematic analysis in psychology. Qual Res Psychol.

[27] (2020). NVivo. QSR International.

[28] Morton LM, Cahill J, Hartge P (2006). Reporting participation in epidemiologic studies: a survey of practice. Am J Epidemiol.

[29] Mahalingaiah S, Fruh V, Rodriguez E, Konanki SC, Onnela JP, de Figueiredo Veiga A, Lyons G, Ahmed R, Li H, Gallagher N, Jukic AM, Ferguson KK, Baird DD, Wilcox AJ, Curry CL, Suharwardy S, Fischer-Colbrie T, Agrawal G, Coull BA, Hauser R, Williams MA (2022). Design and methods of the Apple Women's Health Study: a digital longitudinal cohort study. Am J Obstet Gynecol.

[30] Böhm AK, Jensen ML, Sørensen MR, Stargardt T (2020). Real-world evidence of user engagement with mobile health for diabetes management: longitudinal observational study. JMIR Mhealth Uhealth.

[31] Holmes CJ, Sherman SR, Hornikel B, Cicone ZS, Wind SA, Esco MR (2020). Compliance of self-measured HRV using smartphone applications in collegiate athletes. J High Technol Manag Res.

[32] Huckins JF, daSilva AW, Wang W, Hedlund E, Rogers C, Nepal SK, Wu J, Obuchi M, Murphy EI, Meyer ML, Wagner DD, Holtzheimer PE, Campbell AT (2020). Mental health and behavior of college students during the early phases of the COVID-19 pandemic: longitudinal smartphone and ecological momentary assessment study. J Med Internet Res.

[33] Mundnich K, Booth BM, L'Hommedieu M, Feng T, Girault B, L'Hommedieu J, Wildman M, Skaaden S, Nadarajan A, Villatte JL, Falk TH, Lerman K, Ferrara E, Narayanan S (2020). TILES-2018, a longitudinal physiologic and behavioral data set of hospital workers. Sci Data.

[34] Avram R, Tison GH, Aschbacher K, Kuhar P, Vittinghoff E, Butzner M, Runge R, Wu N, Pletcher MJ, Marcus GM, Olgin J (2019). Real-world heart rate norms in the Health eHeart study. NPJ Digit Med.

[35] Perez MV, Mahaffey KW, Hedlin H, Rumsfeld JS, Garcia A, Ferris T, Balasubramanian V, Russo AM, Rajmane A, Cheung L, Hung G, Lee J, Kowey P, Talati N, Nag D, Gummidipundi SE, Beatty A, Hills MT, Desai S, Granger CB, Desai M, Turakhia MP, Apple Heart Study Investigators (2019). Large-scale assessment of a smartwatch to identify atrial fibrillation. N Engl J Med.

[36] Brower J, LaBarge MC, White L, Mitchell MS (2020). Examining responsiveness to an incentive-based mobile health app: longitudinal observational study. J Med Internet Res.

[37] Spartano NL, Lin H, Sun F, Lunetta KL, Trinquart L, Valentino M, Manders ES, Pletcher MJ, Marcus GM, McManus DD, Benjamin EJ, Fox CS, Olgin JE, Murabito JM (2019). Comparison of on-site versus remote mobile device support in the Framingham heart study using the health eHeart study for digital follow-up: randomized pilot study set within an observational study design. JMIR Mhealth Uhealth.

[38] van Dijk WJ, Saadah NH, Numans ME, Aardoom JJ, Bonten TN, Brandjes M, Brust M, le Cessie S, Chavannes NH, Middelburg RA, Rosendaal F, Visser LG, Kiefte-de Jong J (2021). COVID RADAR app: description and validation of population surveillance of symptoms and behavior in relation to COVID-19. PLoS One.

[39] Menni C, Valdes AM, Freidin MB, Sudre CH, Nguyen LH, Drew DA, Ganesh S, Varsavsky T, Cardoso MJ, El-Sayed Moustafa JS, Visconti A, Hysi P, Bowyer RC, Mangino M, Falchi M, Wolf J, Ourselin S, Chan AT, Steves CJ, Spector TD (2020). Real-time tracking of self-reported symptoms to predict potential COVID-19. Nat Med.

[40] Enzenbach C, Wicklein B, Wirkner K, Loeffler M (2019). Evaluating selection bias in a population-based cohort study with low baseline participation: the LIFE-Adult-Study. BMC Med Res Methodol.

[41] (2021). UK Consumer Digital Index 2021. Lloyds Bank.

[42] Engelhard MM, Patek SD, Sheridan K, Lach JC, Goldman MD (2017). Remotely engaged: lessons from remote monitoring in multiple sclerosis. Int J Med Inform.

[43] Huang F, Chang P, Hou IC, Tu MH, Lan CF (2015). Use of a mobile device by nursing home residents for long-term care comprehensive geriatric self-assessment: a feasibility study. Comput Inform Nurs.

[44] Renfrew ME, Morton DP, Northcote M, Morton JK, Hinze JS, Przybylko G (2021). Participant perceptions of facilitators and barriers to adherence in a digital mental health intervention for a nonclinical cohort: content analysis. J Med Internet Res.

[45] Rodriguez Hermosa JL, Fuster Gomila A, Puente Maestu L, Amado Diago CA, Callejas González FJ, Malo De Molina Ruiz R, Fuentes Ferrer ME, Álvarez Sala-Walther JL, Calle Rubio M (2020). Compliance and utility of a smartphone app for the detection of exacerbations in patients with chronic obstructive pulmonary disease: cohort study. JMIR Mhealth Uhealth.

[46] Reade S, Spencer K, Sergeant JC, Sperrin M, Schultz DM, Ainsworth J, Lakshminarayana R, Hellman B, James B, McBeth J, Sanders C, Dixon WG (2017). Cloudy with a chance of pain: engagement and subsequent attrition of daily data entry in a smartphone pilot study tracking weather, disease severity, and physical activity in patients with rheumatoid arthritis. JMIR Mhealth Uhealth.

[47] Weerts ZZ, Heinen KG, Masclee AA, Quanjel AB, Winkens B, Vork L, Rinkens PE, Jonkers DM, Keszthelyi D (2020). Smart data collection for the assessment of treatment effects in irritable bowel syndrome: observational study. JMIR Mhealth Uhealth.

[48] COVID Oximetry at home. National Health Service England.

[49] Greenhalgh T, Knight M, Inda-Kim M, Fulop NJ, Leach J, Vindrola-Padros C (2021). Remote management of covid-19 using home pulse oximetry and virtual ward support. BMJ.

[50] Ku JP, Sim I (2021). Mobile Health: making the leap to research and clinics. NPJ Digit Med.

[51] (2016). Monitoring and evaluating digital health interventions: a practical guide to conducting research and assessment. World Health Organization.

[52] Murray E, Burns J, May C, Finch T, O'Donnell C, Wallace P, Mair F (2011). Why is it difficult to implement e-health initiatives? A qualitative study. Implement Sci.

