# Supplementary material for: Engagement With mHealth COVID-19 Digital Biomarker Measurements in a Longitudinal Cohort Study: Mixed Methods Evaluation
Source: J Med Internet Res. 2023 Jan 13;25:e40602. doi: 10.2196/40602 (PMC9842396; doi:10.2196/40602)
Supplement: Multimedia Appendix 1 [file jmir_v25i1e40602_app1.docx]

Multimedia Appendix 1

Methods:

Fenland cohort

The Fenland cohort study was designed to investigate the interaction between environmental and genetic factors in determining obesity, type 2 diabetes and related metabolic disorders. Participants were excluded at baseline if they had a known diagnosis of diabetes, a terminal illness with a prognosis of less than one year, psychotic illness, were pregnant or lactating or unable to walk unaided. Between 2005-2015, 12,435 participants took part in phase 1 of the study and 7,795 participants took part in phase 2. No exclusions were applied at phase 2. At both phases, participants attended a clinical visit at one of the Cambridgeshire research sites where physiological measures and questionnaires were completed and blood samples taken.

Measurements

IMD measures relative levels of area deprivation among the 32,844 LSOAs in England and is calculated based on 7 domains of deprivation which includes income, employment, health deprivation and disability, education and skills training, crime, barriers to housing and services and living environment. We categorised IMD score into a two deprivation categories where the high deprivation category was those with an IMD score in the lowest 3 deciles nationally, the most 30% deprived areas nationally and the low deprivation category the remaining 70%. The 10 rural-urban classification categories for output areas were grouped into two categories; urban and rural.

Ethnicity was categorised as two categories; white and non-white (which included the combined categories of South Asian, East Asian, Black and Other). Smoking was classified as current smoker, ex-smoker and non-smoker. Participants were asked to rate their health as poor, fair good or excellent. They were also asked how well they were feeling physically right now; those who reported that they were well enough to do all of usual activities or most of usual activities were categorised as “good”; those who reported either they had stopped doing everything they usually do or felt unwell but could still do some of usual daily activities were classified as “poor/moderate”. Body weight in kilograms (kg) was self-reported in those participants who had access to weighing scales at home. Height in centimetres (cm) was measured by a trained member of the study team during the Fenland Study at both Phases 1 and 2. Body mass index (BMI) (weight in kg / height in metres^2^ (m^2^)) was derived.

Participants were asked to self-report existing health conditions from a list provided and for conditions not listed, to add as free text. All free text was coded into condition categories by two clinicians independently based on International Classification of Diseases and Related Health Problems (ICD) groupings. All health conditions reported were aggregated to 30 health condition categories based on ICD hierarchy classification. The most prevalent five categories in the study are included in these analyses; high blood pressure, asthma, anxiety, depression, and diseases of the musculoskeletal system and connective tissue (musculoskeletal).

The physical activity questionnaire contains questions about physical activity in four domains: at home, at work, commuting and during leisure time. To compute physical activity energy expenditure (PAEE), time spent in activities were multiplied by the metabolic cost of each activity obtained from the physical activity compendium, to provide metabolic equivalents (METs) [12]. These were summated across all domains as total PAEE. If reported time spent in activities was greater than 18 hours (assuming 6 hours sleep), all reported durations of activity were scaled back to 18 hours. PAEE was expressed in kJ/kg/day where 1 MET equates to 71 J/min/kg.

Anxiety was measured using the Generalised Anxiety Disorder questionnaire (GAD-7), a validated self-report tool widely used to assess anxiety in community and clinical samples [13]. Similar to PHQ-8, GAD-7 assesses symptoms occurring in the past two weeks; item rating is also identical (total score 0-21). A score of ≥10 identifies generalized anxiety disorder (GAD) with sensitivity 89%, specificity 82%.

Depression was measured using the Patient Health Questionnaire (PHQ-8), a validated self-report tool widely used to assess depression in community and clinical samples including UK primary care [14]. PHQ-8 assesses depressive symptoms occurring in the past two weeks. Each item is rated as not present (0), several days (1), more than half the days (2), and nearly every day (3), giving a total depression symptom score of 0-24. Using established thresholds, the score can provide categorical outcome of current depression [14] (score ≥10) and has been used in large epidemiological studies to define current depression [15].

Perceived stress was measured using the Perceived Stress Scale (PSS), which is one of the most widely used validated self-report tools for assessing psychological stress in community and clinical samples [16]. PSS assesses stress over the past month. Items are designed to measure how unpredictable, uncontrollable, and overloaded respondents find their lives. PSS scores are obtained by reversing responses (e.g., 0 = 4, 1 = 3, 2 = 2, 3 = 1 & 4 = 0) to the four positively stated items (items 4, 5, 7, & 8) and then summing across all 10 items, with higher scores indicating higher psychological stress.

Dried blood spot samples collected remotely by participants were analysed for SARS-CoV-2 IgG antibodies using a commercial enzyme linked-immunosorbent assay (ELIZA) targeting Spike (S2) and Nucleoprotein (N) from SARS-CoV-2 (Omega Diagnostics, UK), interfacing with the semi-quantitative Omega/Mologic COVID-19 IgG assay [11]. Results were classified into two categories; positive or negative (negative or borderline results). Baseline IgG antibody results were used to ascertain whether exposure to the SARS-CoC-2 virus influenced initial engagement level. Results from all antibody tests during the study were also combined to determine if a positive antibody test was associated with continued engagement. Participants categorised as positive if any result was positive at any of the three time points (0, 3 and 6 months) or negative if the result was not positive at any of the three time points.
