# Supplementary material for: Engagement With mHealth COVID-19 Digital Biomarker Measurements in a Longitudinal Cohort Study: Mixed Methods Evaluation
Source: J Med Internet Res. 2023 Jan 13;25:e40602. doi: 10.2196/40602 (PMC9842396; doi:10.2196/40602)
Supplement: Multimedia Appendix 2 [file jmir_v25i1e40602_app2.docx]

Multimedia Appendix 2. Supplementary tables

**Supplementary Table 1: Participant recruitment to the qualitative interviews**

|  | **Invited** | **Accepted** | **Consented** | **Interviewed** |
| --- | --- | --- | --- | --- |
| Web-only | 142 | 15 | 13 | 13 |
| App sub-study | 155 | 25 | 23 | 22 |
| Total | 297 | 40 | 36 | 35 |

**Supplementary Table 2: Baseline characteristics of participants in qualitative study by consent status: App sub-study or Web-only**

|  | **App sub-study n=22** | **Web-only n=13** |
| --- | --- | --- |
| Men, n (%) | 10 (45.5) | 7 (53.8) |
| Age category |  |  |
| <60 | 8 (36.4) | 6 (46.2) |
| >60 | 14 (63.6) | 7 (53.8) |
| higher degree, n (%) |  |  |
|  | 12 (54.5) | 5 (38.5) |
| Median level of engagement with app modules |  |  |
| 0 (no interaction) | 7 (31.8) |  |
| 1 (1-2 times/week) | 7 (31.8) |  |
| 2 (3+ times/week) | 8 (36.4) |  |
