# Supplementary figures and images for: Engagement With mHealth COVID-19 Digital Biomarker Measurements in a Longitudinal Cohort Study: Mixed Methods Evaluation
Source: J Med Internet Res. 2023 Jan 13;25:e40602. doi: 10.2196/40602 (PMC9842396; doi:10.2196/40602)

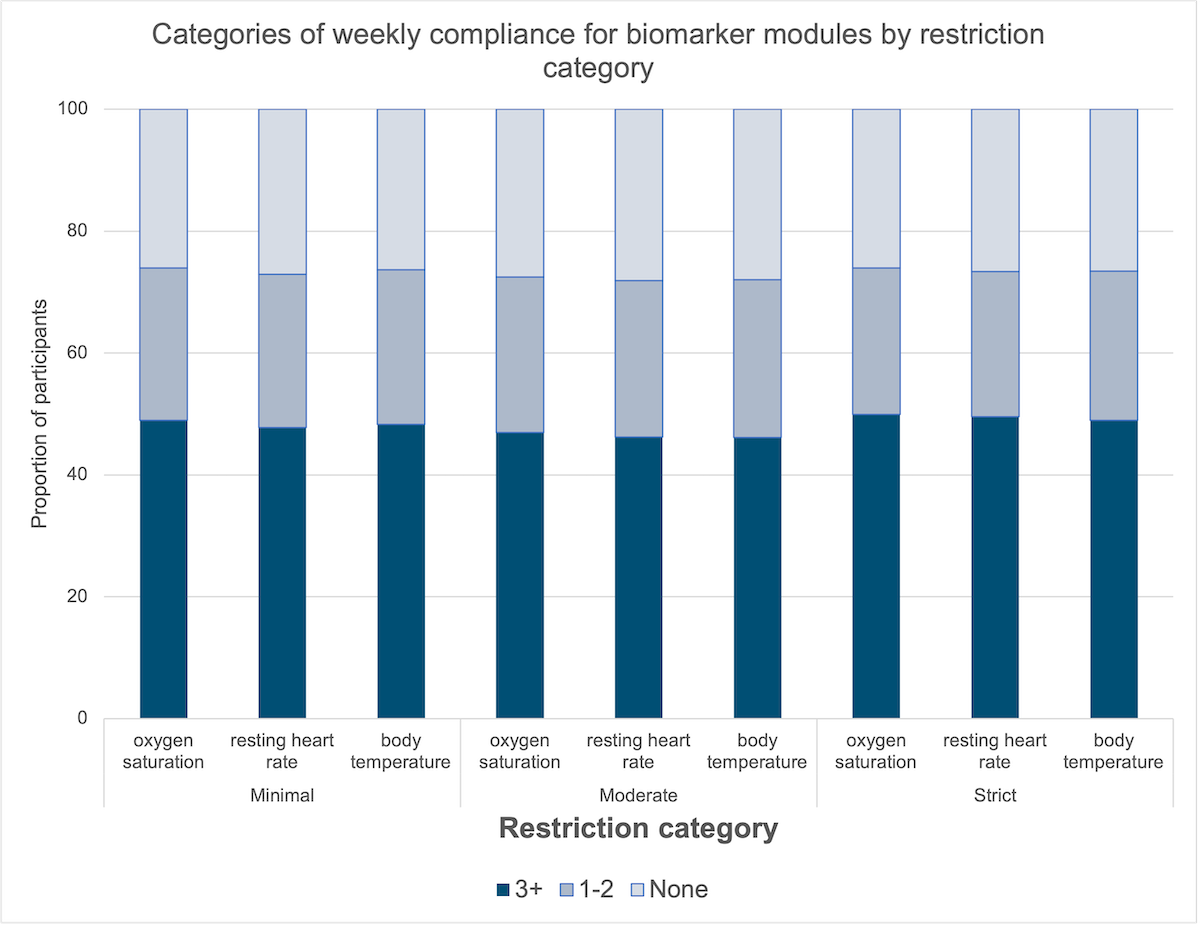

Supplement: Multimedia Appendix 3 [file jmir_v25i1e40602_app3.png]
